# Validity of an AI-Assisted Dietary Recording Application for Family-Based Nutritional Management in Young Patients with Anorexia Nervosa

**DOI:** 10.3390/nu18040708

**Published:** 2026-02-23

**Authors:** Nao Shiraishi, Rieko Kawase, Haruka Ogawa, Tatsuo Akechi

**Affiliations:** 1 Department of Psychiatry and Cognitive-Behavioral Medicine, Graduate School of Medical Sciences, Nagoya City University, 1 Kawasumi, Mizuho-cho, Mizuho-ku, Nagoya 467-8601, Japan; 2 Clinical Nutritional Management Unit, Nagoya City University Hospital, 1 Kawasumi, Mizuho-cho, Mizuho-ku, Nagoya 467-8601, Japan

**Keywords:** artificial intelligence, caregivers, dietary assessment, eating disorders, mobile applications, nutrition therapy, validation study

## Abstract

**Background**: Evidence regarding the validity of digital dietary recordings in the family-based nutritional management of anorexia nervosa (AN) remains limited. This study evaluated the validity of an AI-assisted dietary recording application (app) used by the parents of young patients with AN, with total energy intake as the primary outcome and major nutrient intake as the secondary outcome. **Methods**: During hospital leave prior to discharge, one day of dietary intake was recorded by parents using the app based on meal photographs. The energy and nutrient intakes estimated by the app were compared with a registered dietitian using visual estimation of the photographs. Differences were examined using the Wilcoxon signed-rank test, associations were assessed using Spearman’s correlation coefficients, and agreement was evaluated using Bland–Altman analysis. Sensitivity analyses excluding extreme outliers were performed. **Results**: Thirty female inpatients with AN (mean age: 14.8 ± 2.9 years) and their mothers participated. The median total energy intake did not differ significantly between the app and reference method (2462 vs. 2439 kcal/day). Moderate to high correlations were observed for total energy (*ρ* = 0.62) and major nutrient intakes. The app tended to overestimate these intakes; however, Bland–Altman analyses indicated no systematic bias. Exclusion of two outliers strengthened correlations for total energy intake (*ρ* = 0.74) and narrowed the limits of agreement. **Conclusions**: The app demonstrated an acceptable agreement for estimating energy and major nutrient intake in the therapeutic context of AN. Careful attention to dish type and portion size may further support its clinical use.

## 1. Introduction

Anorexia nervosa (AN) is a severe and potentially life-threatening eating disorder primarily affecting adolescents and young adults, particularly females. With an estimated lifetime prevalence of 0.8–6.3% in women [1], its incidence peaks during adolescence, as approximately 40% of cases occur in 15- to 19-year-old females [2]. In recent decades, the incidence and prevalence of eating disorders, including AN, have steadily increased worldwide, highlighting a growing public health concern [3,4]. AN often leads to extreme malnutrition and medical complications requiring intensive treatment, and has one of the highest mortality rates among mental disorders, with approximately 5% of patients dying from organ failure or suicide [5]. These epidemiological realities highlight the need for enhanced therapeutic strategies for AN, especially those supported by digital health technologies that facilitate nutritional rehabilitation for the affected youth.

Digital health interventions have been explored for individuals with AN, including smartphone applications (apps) and Internet-based programs aimed at supporting weight restoration and preventing relapses. A pilot trial of a smartphone aftercare app reported high patient adherence and acceptability but no significant improvements in body mass index (BMI) and eating disorder symptoms [6]. Internet-based relapse prevention demonstrated no significant increase in weight gain and a significant decrease in eating disorder symptoms among patients discharged from hospital for AN [7]. However, patient-focused digital approaches may be limited by their reliance on self-monitoring. This reliance is problematic given that patients with AN are prone to misjudging portion sizes and overreporting their food intake [8]. Moreover, existing digital interventions have focused almost exclusively on the patients themselves, with minimal integration with family management. Family members, particularly parents, play a pivotal role in the nutritional restoration of adolescents with AN. Family-Based Treatment (FBT) is currently considered the most effective evidence-based treatment for adolescent AN [9,10]. In FBT, parents are expected to take primary responsibility for nutritional restoration because the patient’s eating behavior is strongly influenced by illness. However, little is known about the extent to which family caregivers use digital health technologies to assist in their children’s nutritional restoration.

The present study was designed to address this gap by leveraging a dietary recording app for family-based nutritional management of AN. We selected the Asken app, a widely used Japanese AI-assisted dietary recording tool, because of its demonstrated validity in estimating nutrient intake compared to traditional weighed dietary records [11]. In evaluations of dietary recording apps, the Asken app also showed strong concordance with paper-based dietary records used as the reference method, with a median Spearman correlation coefficient of 0.80 for nutrient intake [12]. Notably, recent clinical trials have incorporated the Asken app into nutritional interventions for other medical conditions, including dietary therapy for type 2 diabetes [13] and LDL-cholesterol management for familial hypercholesterolaemia [14]. Accordingly, this study aimed to evaluate the validity of the Asken application as an AI-assisted dietary recording tool in the therapeutic context of AN. Given that energy intake constitutes a central component of nutritional management in AN, the primary objective was to examine the validity of the app’s estimated total energy intake compared to the reference method assessed by a registered dietitian. As a secondary objective, we further assessed the validity of major macronutrient intake (i.e., protein, fat, and carbohydrates). By focusing on these nutritional parameters, this study explored whether an AI-assisted dietary recording application could provide sufficiently accurate intake estimates to support family-based nutritional management in young patients with AN.

## 2. Materials and Methods

### 2.1. Participants

The study participants were patients with AN admitted to the psychiatric ward of Nagoya City University Hospital and their parents. The purpose of admission was to undergo nutritional therapy to restore the severely low body weight. The inclusion criteria were inpatients who (1) aged between 10 and 24 years; (2) diagnosed with AN according to the Diagnostic and Statistical Manual of Mental Disorders, Fifth Edition [15]; (3) BMI of less than 17; (4) had pre-admission meals provided by their participating parents. This study excluded inpatients who were (1) male, (2) pregnant, or (3) requiring nutritional therapy for conditions other than AN. Male patients were excluded to maintain a more homogeneous study sample as sex-related differences in body composition and nutritional requirements introduce additional variability that might affect the validation of dietary intake estimates.

Eligible participants were recruited once inpatients had reached a BMI of 15.5, prior to consuming meals during hospital leave in preparation for discharge. The BMI threshold was set with reference to the manual for the physical management of AN [16], which defines a BMI of 16.0 as the minimum weight required for safe outpatient treatment. After receiving a thorough explanation of the research purpose and methods, written informed consent was obtained from all the participants. The study was approved by the Institutional Review Boards of Nagoya City University Graduate School of Medical Sciences and Nagoya City University Hospital (protocol code: 60-20-0060) and was conducted in accordance with the principles of the Declaration of Helsinki.

### 2.2. Dietary Record

During hospital leave, the patients’ parents were asked to record their dietary intake using the Asken app installed on an iPad provided by the hospital for research purposes. Before lending the iPad, parents received a brief 20 min orientation on app use, as outlined below.

**Figure 1:**
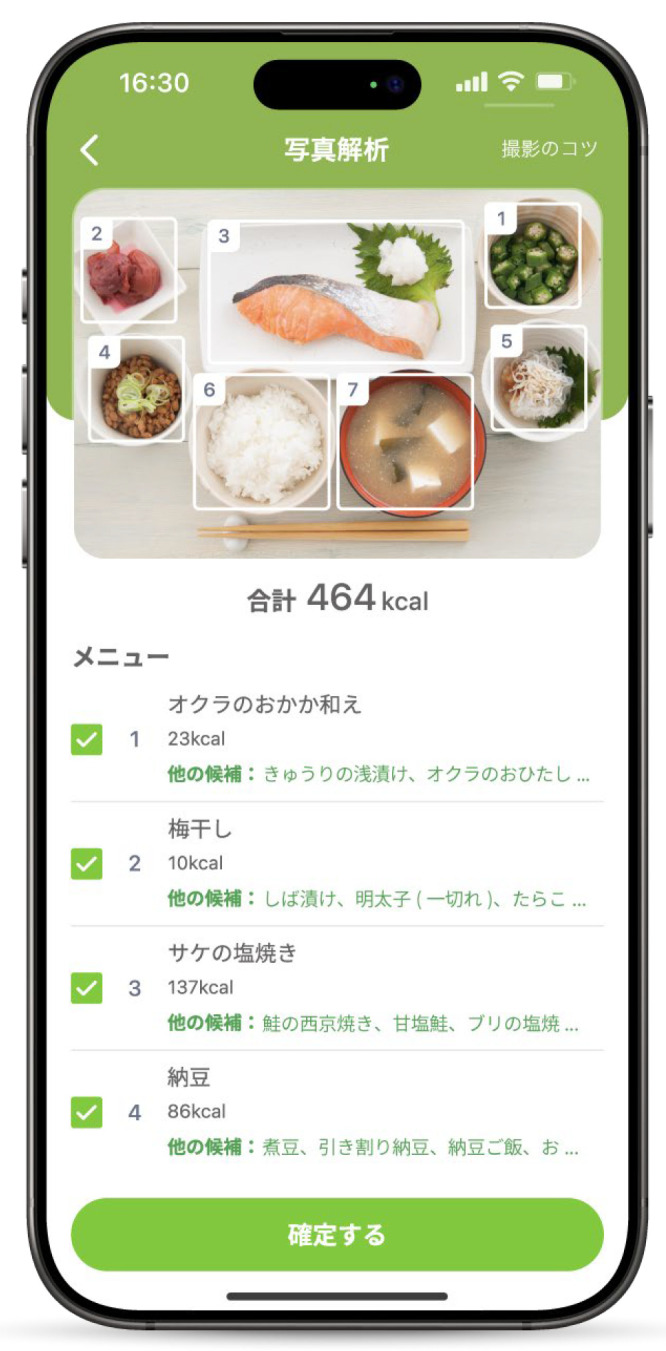
AI-assisted food item recognition and selection process in the AI-assisted dietary recording app.

1. Parents were instructed to prepare meals that were typical for their household, while also reflecting on what they considered necessary to support weight restoration after discharge. When they prepared a meal (breakfast, lunch, or dinner), each meal was photographed with the iPad before eating, ensuring that the patient did not participate in meal preparation. Parents were also instructed to photograph only a single serving and place a ruler alongside the dishes, allowing portion sizes to be accurately estimated using the photographs as a reference [17].
2. The photographs were analyzed using the AI assessment system embedded in the app. The system automatically displayed the candidate food items for each dish (Figure 1). If the suggested items were correct, the parents confirmed them. If an item was incorrect, they selected the correct item from among the options displayed. When no appropriate option was available, the participants searched for foods by entering their names into the search box. The Asken app is supported by a comprehensive food database containing more than 150,000 menu items. Parents could search for and confirm foods using commercial products, common menus, or individual ingredient names.
3. Upon returning from hospital leave, parents returned the iPad to the ward staff. The analysis results obtained from the app were then used to collect data on energy intake (kcal/day) and the following 13 nutrient intakes: protein (g/day), fat (g/day), saturated fatty acids (SFAs, g/day), carbohydrates (g/day), dietary fiber (g/day), calcium (mg/day), iron (mg/day), vitamin A (µg/day), vitamin E (mg/day), vitamin B1 (mg/day), vitamin B2 (mg/day), vitamin C (mg/day), and sodium (g/day).

### 2.3. Reference Method

A registered dietitian served as an independent evaluator. She had 13 years of professional experience as a registered dietitian, including 9 years of experience in the nutritional rehabilitation of patients with eating disorders, mainly AN. The dietitian was blinded to the results obtained from the app. She received encrypted meal photographs and analyzed them using a secure computer within the university hospital network. She estimated energy and 13 nutrient intakes through a visual assessment of food types and portion sizes using a ruler alongside dishes in the photographs and referencing the Standard Tables of Food Composition in Japan (2020 edition) [18].

### 2.4. Statistical Analysis

The required sample size was determined based on correlation analysis to evaluate the similarity in total energy intake between the app and the dietitian-assessed reference method. Referring to the study by Matsuzaki et al. [11], we assumed a correlation coefficient of 0.6, with a two-sided α of 0.05 and a power of 0.90. This calculation indicated that 25 participants were required, accounting for an anticipated 20% of the missing data. The final target sample size was set to 30. Missing data were to be excluded from subsequent analyses.

Descriptive statistics are presented as the median and interquartile range (IQR) for energy and nutrient intake estimated by the app and the reference method. The differences between the two methods were evaluated using the Wilcoxon signed-rank test (two-sided, *α* = 0.05). The association between the two methods was then quantified through Spearman’s rank correlation coefficient (*ρ*), with 95% confidence intervals (95% CIs) estimated by nonparametric bootstrap resampling (10,000 replicates). In addition to unadjusted correlations, energy-adjusted correlations were quantified using the residual (energy-adjustment) method: residuals from a linear regression of each variable on total energy intake were obtained for both methods, and Spearman’s *ρ* values with 95% CIs were estimated between the corresponding residuals [19]. The strength of Spearman’s *ρ* was interpreted as follows: values of 0–0.29 indicated negligible correlation, 0.30–0.49 low correlation, 0.50–0.69 moderate correlation, 0.70–0.89 high correlation, and 0.90–1.00 very high correlation [20]. The agreement between the two methods was further evaluated using Bland–Altman analysis [21]. For each variable, the mean difference (bias) between the two methods and the 95% limits of agreement (LOAs; mean difference ± 1.96 × standard deviation of the differences) were calculated. Bland–Altman plots were created to visualize the agreement and identify any systematic bias or trends across the range of measurements.

Sensitivity analysis was conducted in cases where participants showed large discrepancies in energy intake between the app and the reference method. For each participant, the difference in the total energy intake between the two methods was calculated and illustrated using a bar chart. A threshold of ±1000 kcal was applied to highlight cases with notably higher or lower estimates from the application compared with the reference method. Based on the manual for the physical treatment of AN [16], this threshold corresponds to the additional energy intake required to achieve a weight gain of approximately 1 kg/week. One thousand kcal is regarded as the upper limit of the acceptable physical burden per day during AN treatment. After excluding participants whose energy intake differences exceeded this threshold, the same statistical procedures as described above—namely, the Wilcoxon signed-rank test, Spearman’s rank correlation, and Bland–Altman analysis—were repeated to examine whether the exclusion of outliers affected the overall agreement between the two methods.

All statistical analyses were performed using Python (version 3.12.11; Python Software Foundation, Wilmington, DE, USA) on Google Colaboratory (Google LLC, Mountain View, CA, USA). A large language model (ChatGPT, version 5.2; OpenAI) was used for English language editing of the manuscript, including improvements to grammar, wording, and clarity.

## 3. Results

### 3.1. Participant Characteristics

A total of 31 female patients with AN and their mothers were enrolled between September 2020 and July 2025. No fathers were enrolled during the study period. One parent–child pair was excluded from the analyses because the mother photographed meals served to the entire family. All 30 participants provided complete dietary records. The sociodemographic and clinical characteristics are summarized in Table 1. All patients were students aged 10–22 years, with a mean illness duration of 12.3 months. The restrictive subtype accounted for the majority of the patients (86.7%). During the current admission, the mean BMI increased from 12.7 at admission to 16.4 at the time of dietary recording over an average hospital stay of 74.4 days. All involuntarily admitted patients (40%) were converted to voluntary admissions prior to dietary recording. The mothers were aged 38–56 years. Regarding education, 43.3% and 36.7% had completed a two-year college and university education, respectively. One-third of the mothers were regularly employed, whereas an additional one-third reported irregular employment. All mothers were married, and 93.3% had one to three children. Only 10% of the mothers received treatment for mental health problems.

### 3.2. Differences and Associations Between the App and the Reference Method

The energy and major nutrient intakes estimated by the app were generally comparable to those obtained by the reference method (Table 2). Total energy intake did not differ significantly between the two methods (median: 2462 vs. 2439 kcal/day; *p* = 0.903). No significant differences were observed in the protein or carbohydrate intake between the two methods (*p* = 0.452 and *p* = 0.061, respectively). In contrast, the app yielded significantly higher values for 11 nutrients: fat, saturated fatty acids, dietary fiber, calcium, iron, vitamins A, E, B1, B2, C, and sodium (all *p* < 0.05).

In the unadjusted analyses, Spearman’s correlation coefficients (*ρ*) indicated moderate to high associations between the app and the reference method across most nutrients (*ρ* = 0.56–0.79). After adjustment for energy intake, the associations remained moderate to high for 10 of the 13 nutrients (*ρ* = 0.50–0.72), except for SFAs and vitamins A and E.

### 3.3. Agreement Between the App and the Reference Method

**Figure 2:**
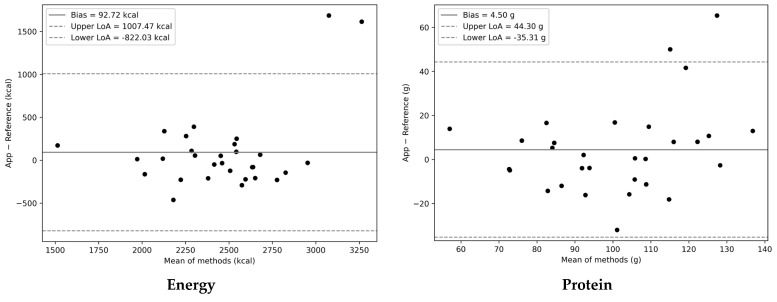

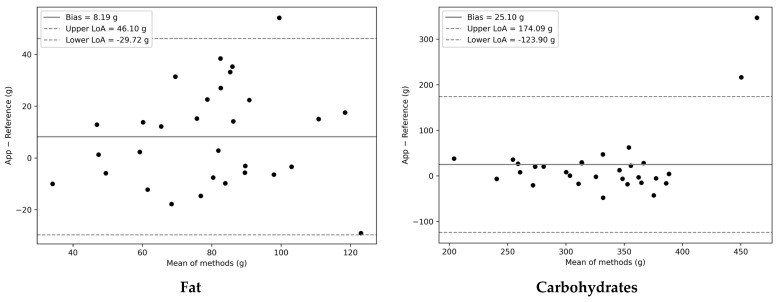
Bland–Altman plots for energy intake and the major nutrient intakes (protein, fat, and carbohydrates) comparing the app with the reference method (*n* = 30). The solid line shows the bias, and the dashed lines represent the 95% limits of agreement (LoAs).

Bland–Altman analysis was conducted to assess the agreement between the app and the reference method for energy and nutrient intake. As shown in Figure 2, the mean bias between the two methods was generally small for total energy, protein, fat, and carbohydrate intakes, with most of the data points falling within the 95% limits of agreement (LoAs). No systematic trends were evident across the range of measurements, indicating overall good agreement between the two methods for energy and major nutrients. For the remaining nutrients, SFAs, dietary fiber, calcium, iron, vitamins A, E, B1, B2, C, and sodium, the Bland–Altman plots revealed no apparent systematic bias, with most observations falling within the 95% LoAs (Supplementary Figure S1).

However, the 95% LoA was relatively wide for several nutrients—particularly for total energy (−822.03 to 1007.47 kcal/day)—suggesting the presence of considerable interindividual variability among participants. A few participants showed marked deviations from the mean bias, which may have affected overall agreement.

### 3.4. Individual Differences in Energy Intake

**Figure 3:**
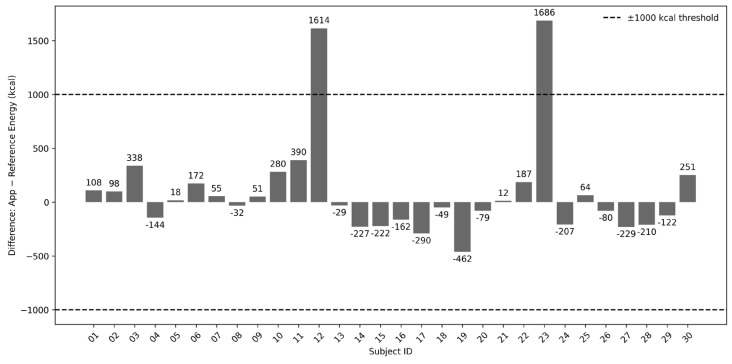
Individual differences in total energy intake between the app and the reference method.

Figure 3 illustrates the individual differences in the total energy intake between the two methods. Two participants showed differences exceeding 1000 kcal/day, suggesting a potential overestimation by the app compared with the reference method. The following cases describe the main misclassifications of dish type and portion size for participants ID12 and ID23. In the former, lunch included a discrepancy, in which a plain rolled omelet was recorded in the app as an omelet rice dish, resulting in an overestimation of 522 kcal. For dinner, although “ramen noodles” was correctly selected, the portion size was entered in the app as a full serving instead of the actual smaller amount, leading to an additional overestimation of 261 kcal. Overall, the portion sizes entered into the app did not match those shown in the photographs. In the latter case, as a dinner dish, the app selected a type of meat with a higher estimated fat content than the reference method, resulting in an overestimation of 953 kcal. For lunch, “tenshinhan,” a Chinese-style rice dish with egg and sauce, was recorded in the app as a restaurant-style portion that contained larger amounts of egg and oil, producing a further overestimation of 759 kcal.

### 3.5. Sensitivity Analysis

**Figure 4:**
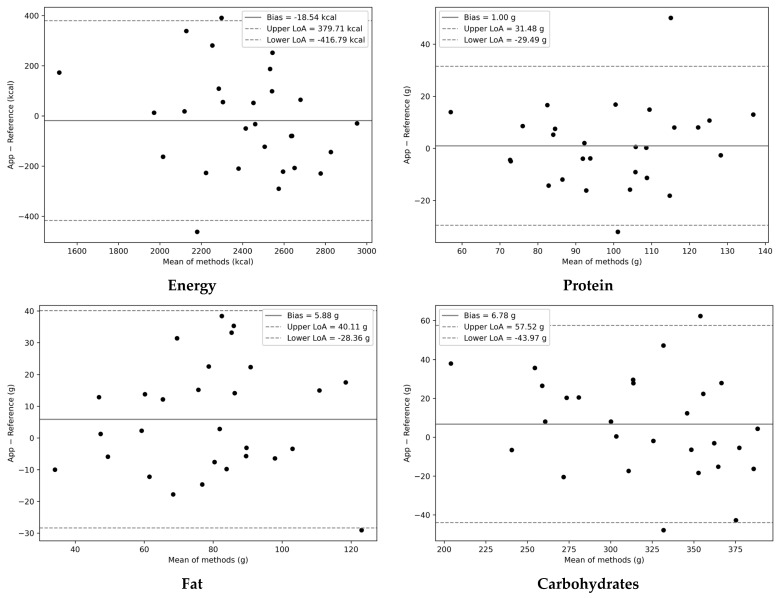
Sensitivity analysis of Bland–Altman plots for energy intake and the major nutrient intakes (protein, fat, and carbohydrates) comparing the app with the reference method (*n* = 28). The solid line indicates the bias, and the dashed lines represent the 95% limits of agreement (LoAs).

As shown in Table 3, the exclusion of participants ID12 and ID23 improved the overall consistency between the app and the reference methods. The median energy intake estimated using the app remained comparable to that estimated using the reference method (2445 vs. 2439 kcal/day; *p* = 0.614), which is in line with the findings of the primary analysis. Moreover, the correlations between the two methods became stronger for most nutrients, with unadjusted Spearman’s *ρ* ranging from 0.59 to 0.84 and energy-adjusted *ρ* ranging from 0.53 to 0.71. In particular, for total energy—a central parameter in the treatment of AN—Spearman’s correlation coefficient increased from 0.62 (95% CI: 0.32–0.82) to 0.74 (95% CI: 0.49–0.89), indicating stronger consistency in ranking between the two methods. Correspondingly, Bland–Altman analysis revealed that the 95% LoA narrowed, with the lower limit improving from −822.03 kcal to −416.79 kcal and the upper limit from 1007.47 kcal to 379.71 kcal. Figure 4 and Supplementary Figure S2 show no evident systematic trend across the range of measurements, thereby confirming a stable agreement between the two methods after the exclusion of outliers.

## 4. Discussion

### 4.1. Summary of Main Findings

This study evaluated the validity of an AI-assisted dietary recording application in comparison with dietitian-assessed visual estimation in young inpatients with AN receiving nutritional therapy. The total energy intake estimated by the app did not significantly differ from that obtained by the reference method (median: 2462 vs. 2439 kcal/day) and showed a moderate correlation (*ρ* = 0.62). Energy-adjusted analyses also confirmed moderate-to-high correlations for 10 of the 13 nutrients. Although the app tended to overestimate energy and nutrient intakes, sensitivity analyses excluding outliers strengthened the validity of results, with an improved correlation for total energy intake (*ρ* = 0.74) and major nutrients. Consistently, Bland–Altman analysis demonstrated a narrower LoA for energy and major nutrients after the exclusion of outliers, indicating an enhanced agreement between the two methods. These findings suggest that the app can estimate the total energy and major nutrient intake with an accuracy comparable to that of a trained dietician.

### 4.2. Interpretation of Study Findings

Although evidence regarding the accuracy of parent-assessed dietary records for children with AN is limited, an overestimation trend has been documented in dietary self-reports among individuals with the illness. Previous studies [22,23] indicated that patients with AN tend to overestimate their energy intake. Hadigan et al. [22] reported that women with AN (*n* = 30) had a higher caloric intake in their diet history than that measured during the observation period (mean: 1602 vs. 1289 kcal/day; *p* < 0.05). Similarly, Schebendach et al. [23] found that patients with weight-restored AN (*n* = 12) overestimated their energy intake by approximately 16% during a controlled meal challenge. Despite the limited generalizability of these findings owing to small sample sizes and laboratory-based observations, overestimation in self-reports could be attributable to the clinical characteristics of individuals with AN, such as body image distortion, perfectionism, cognitive rigidity [24], and impaired interoceptive awareness [25]. These characteristics are known to bias patients’ perceptions of portion size, caloric content, and internal satiety cues [8]. Participating mothers were instructed to ensure that their daughters did not interfere with the patients’ meal preparation. Nevertheless, mother–daughter interactions may have allowed the patients’ biased perceptions to influence the estimation process, possibly contributing to the app’s overestimation of energy intake.

The overestimation of energy intake might partly reflect parents’ limited experience with tablet-based data entry or their low confidence in using the app. Moreover, family dynamics presumably played a role in the observed overestimation of energy intake, particularly through parental behavior. One hypothesis is that a subset of parents is engaged in accommodating eating disorders by modifying their behavior to reduce their child’s distress [26]. Anderson et al. [27] reported that family caregivers’ anxiety was associated with accommodating behaviors (e.g., bending rules to avoid conflict) in patients with eating disorders. In our study, such accommodating behaviors could partly explain the overreporting of food intake out of sympathy, particularly with the intention of hastening hospital discharge. Another hypothesis is that careless dietary logging results from parental indifference toward their child’s care. Caregiver burdens and burnout can lead to emotional disengagement as a coping mechanism. Parks et al. [28] found that avoidance-based coping styles were more likely to be adopted by mothers who described their caregiving experiences negatively. In our study, some parents may have entered the data inaccurately or without vigilance, potentially contributing to an unintentional overestimation of food intake.

### 4.3. Strengths and Limitations

The present study had several limitations. Firstly, the generalizability of our findings may be limited because the sample consisted exclusively of inpatients with AN undergoing weight restoration at a university hospital. Therefore, our findings may not extend to males, outpatients, patients treated in other clinical settings, or individuals at different stages of illness. Nevertheless, the clinical homogeneity of this sample allowed for a more controlled comparison between the app-based estimates and reference method. Secondly, the reference method relies on the visual estimation of meal photographs by an experienced dietitian. Although this approach has been widely used in dietary assessments [17], it is not the gold standard and is inherently subject to measurement errors, particularly in the estimation of portion size and food content. Such errors may be influenced by photographic conditions, such as angle, distance, and lighting. Therefore, the differences observed between the app and reference methods do not provide definitive evidence of systematic overestimation or underestimation by either method. Thirdly, the estimation of micronutrient intake from meal photographs was uncertain. Visual assessment cannot reliably capture vitamin and mineral contents without weight records or chemical composition data, even when performed by an experienced dietitian. Therefore, the micronutrient results should be considered exploratory. Fourthly, inter-rater reliability was not assessed as the reference evaluation was conducted by an experienced dietitian, which may limit the generalizability of the reference measurements. Fifthly, dietary intake was assessed for only a single day, which did not account for habitual intake or day-to-day variability within individuals [29]. This may limit the generalizability of intake values and their applicability to long-term dietary assessments. However, this study aimed to examine the agreement between the two assessment methods rather than to estimate the usual intake. If both methods assessed the same meals under comparable conditions, a single-day record was considered appropriate. Finally, the app occasionally misclassified dish types or portion sizes, leading to a substantial overestimation for a small number of participants. Nevertheless, sensitivity analysis that excluded these outliers demonstrated that the main findings remained robust.

## 5. Conclusions

This study provides preliminary evidence that AI-based dietary assessment can serve as an alternative to expert evaluation for the clinical management of young patients with AN. The overall level of agreement suggests that the app has potential clinical utility when used with appropriate attention to portion size inputs and dish selection. Further validation studies in broader clinical settings as well as refinement of the assessment system are needed.

## Figures and Tables

**Table 1:** Sociodemographic and clinical characteristics of patients with anorexia nervosa (AN) and their mothers.

Patients (*n* = 30)			Mothers (*n* = 30)		
Age, mean (SD), years	14.8	(2.9)	Age, mean (SD), years	45.9	(4.8)
Current level of education, *n* (%)			Highest level of education, *n* (%)		
Elementary school	4	(13.3)	Compulsory education	2	(6.7)
Junior high school	14	(46.7)	High school	4	(13.3)
High school	7	(23.3)	Two-year college	13	(43.3)
Two-year college	1	(3.3)	University	11	(36.7)
University	4	(13.3)	Occupational status		
AN subtype, *n* (%)			Regular employment	10	(33.3)
Restricting	26	(86.7)	Irregular employment	10	(33.3)
Binge-eating/purging	4	(13.3)	Self-employment	2	(6.7)
Amenorrhea at current admission, *n* (%)			Houseperson	8	(26.7)
Yes	20	(66.7)	Marital status, *n* (%)		
No	2	(6.7)	Married	30	(100.0)
Not yet menarche	8	(26.7)	Unmarried	0	
Duration of illness, mean (SD), months ^1^	12.3	(10.2)	Number of children, *n* (%) ^4^		
Number of hospitalizations due to AN, *n* (%) ^2^			One	5	(16.7)
One	19	(63.3)	Two	14	(46.7)
Two	4	(13.3)	Three	9	(30.0)
Three	5	(16.7)	Four	1	(3.3)
Four	1	(3.3)	Five	1	(3.3)
Five	1	(3.3)	Mental health treatment, *n* (%)		
Admission type at current admission			Yes	3	(10.0)
Voluntary	18	(60.0)	No	37	(90.0)
Involuntary	12	(40.0)			
Duration of hospitalization, mean (SD), days ^3^	74.4	(27.1)			
BMI at current admission, mean (SD)	12.7	(1.5)			
BMI at dietary recording, mean (SD)	16.4	(0.9)			

^1^ Duration of illness: up to the current admission; ^2^ Number of hospitalizations due to AN: total including the current admission; ^3^ Duration of hospitalization: from the current admission to the dietary recording; ^4^ Number of children: total including the patient.

**Table 2:** Differences and Associations in energy and nutrient intakes: the dietary recording app vs. the reference method (*n* = 30).

	AI-Assisted Dietary Recording App		Dietitian-Assessed Reference Method		Test ^1^	Spearman’s Correlation Coefficient			
	Median	IQR	Median	IQR	*p*-Value	*ρ*	95% CI	*ρ*	95% CI
	(Unadjusted) ^2^		(Unadjusted) ^2^			(Unadjusted) ^2^		(Adjusted) ^2^	
Energy (kcal/day)	2462	2306–2619	2439	2231–2667	0.903	0.62	0.32–0.82	—	
Protein (g/day)	98.8	85.5–119.2	97.1	90.0–112.2	0.452	0.62	0.30–0.84	0.53	0.09–0.79
Fat (g/day)	86.0	67.7–101.6	70.7	60.3–87.6	0.047 *	0.63	0.30–0.84	0.63	0.25–0.83
SFAs (g/day)	19.23	14.54–25.35	16.98	11.31–21.44	0.050 *	0.34	−0.05–0.65	0.34	−0.03–0.66
Carbohydrates (g/day)	336.0	293.9–365.3	321.1	284.1–355.0	0.061	0.73	0.42–0.89	0.60	0.15–0.80
Dietary fiber (g/day)	27.6	23.1–35.0	16.1	12.3–21.1	<0.001 ***	0.62	0.28–0.83	0.52	0.18–0.70
Calcium (mg/day)	805	610–1037	704	566–859	0.008 **	0.72	0.45–0.90	0.59	0.14–0.82
Iron (mg/day)	10.9	8.9–14.8	9.0	7.4–11.7	0.002 **	0.69	0.37–0.89	0.50	0.12–0.80
Vitamin A (μg/day)	870	694–1261	677	543–871	<0.001 ***	0.64	0.33–0.85	0.32	−0.06–0.70
Vitamin E (mg/day)	15.8	11.6–18.5	12.1	7.8–15.0	<0.001 ***	0.56	0.18–0.86	0.33	−0.12–0.65
Vitamin B1 (mg/day)	1.55	1.29–1.96	1.32	1.16–1.76	0.036 *	0.70	0.45–0.86	0.50	0.07–0.76
Vitamin B2 (mg/day)	1.97	1.54–2.19	1.68	1.35–2.29	0.010 **	0.79	0.49–0.95	0.54	0.15–0.81
Vitamin C (mg/day)	133	98–175	95	69–146	0.004 **	0.77	0.49–0.91	0.72	0.39–0.86
Sodium (g/day)	11.1	8.9–14.1	9.5	7.4–11.9	0.007 **	0.74	0.47–0.90	0.68	0.38–0.87

SFA: Saturated fatty acid. ^1^ Wilcoxon signed-rank test; ^2^ Values are unadjusted or adjusted for energy. * *p* < 0.05; ** *p* < 0.01; *** *p* < 0.001.

**Table 3:** Sensitivity analysis for differences and associations in energy and nutrient intakes: the app vs. the reference method (*n* = 28).

	AI-Assisted Dietary Recording App		Dietitian-Assessed Reference Method		Test ^1^	Spearman’s Correlation Coefficient			
	Median	IQR	Median	IQR	*p*-value	*ρ*	95% CI	*ρ*	95% CI
	(Unadjusted) ^2^		(Unadjusted) ^2^			(Unadjusted) ^2^		(Adjusted) ^2^	
Energy (kcal/day)	2445	2292–2595	2439	2201–2675	0.614	0.74	0.49–0.89	—	
Protein (g/day)	94.9	85.0–110.9	98.3	87.8–112.8	0.867	0.70	0.40–0.89	0.56	0.09–0.81
Fat (g/day)	84.3	65.4–101.4	73.0	59.0–89.4	0.126	0.69	0.37–0.87	0.68	0.30–0.86
SFAs (g/day)	17.69	14.22–25.06	16.98	12.35–20.11	0.070	0.37	−0.03–0.68	0.35	−0.02–0.70
Carbohydrates (g/day)	328.0	289.3–358.0	321.1	279.1–357.3	0.164	0.84	0.63–0.93	0.62	0.20–0.83
Dietary fiber (g/day)	26.3	23.0–33.6	15.5	11.7–21.0	<0.001 ***	0.59	0.24–0.81	0.53	0.16–0.72
Calcium (mg/day)	773	610–1024	711	541–871	0.023 *	0.78	0.51–0.93	0.59	0.20–0.84
Iron (mg/day)	10.7	8.8–13.1	9.2	7.0–11.8	0.008 **	0.83	0.64–0.93	0.60	0.21–0.85
Vitamin A (μg/day)	870	672–1189	677	532–879	<0.001 ***	0.70	0.39–0.87	0.47	0.00–0.75
Vitamin E (mg/day)	15.1	11.1–18.0	12.1	8.1–15.4	<0.001 ***	0.73	0.44–0.90	0.45	−0.02–0.73
Vitamin B1 (mg/day)	1.50	1.26–1.87	1.26	1.15–1.81	0.090	0.72	0.43–0.88	0.54	0.09–0.79
Vitamin B2 (mg/day)	1.87	1.50–2.15	1.63	1.32–2.26	0.026 *	0.79	0.46–0.96	0.59	0.18–0.83
Vitamin C (mg/day)	126	94–169	90	68–140	0.010 *	0.75	0.44–0.91	0.68	0.37–0.88
Sodium (g/day)	11.0	8.9–13.3	9.3	7.3–11.3	0.003 **	0.71	0.40–0.89	0.71	0.37–0.89

SFA: Saturated fatty acid. ^1^ Wilcoxon signed-rank test; ^2^ Values are unadjusted or adjusted for energy. * *p* < 0.05; ** *p* < 0.01; *** *p* < 0.001.

## Data Availability

The datasets analyzed in this study are available from the corresponding author upon reasonable request, due to privacy and ethical restrictions related to sensitive clinical data.

## Supplementary Materials

The following supporting information can be downloaded at: https://www.mdpi.com/article/10.3390/nu18040708/s1, Figure S1: Bland–Altman plots for nutrient intakes (saturated fatty acids, dietary fiber, calcium, iron, vitamins A, E, B1, B2, C, and sodium) comparing the app with the reference method (*n* = 30); Figure S2: Sensitivity analysis of Bland–Altman plots for nutrient intakes (saturated fatty acids, dietary fiber, calcium, iron, vitamins A, E, B1, B2, C, and sodium) comparing the app with the reference method (*n* = 28).

## Abbreviations

The following abbreviations are used in this manuscript:

AI	Artificial Intelligence
AN	Anorexia Nervosa
BMI	Body Mass Index
CI	Confidence Interval
FBT	Family-Based Treatment
IQR	Interquartile Range
LoAs	Limits of Agreement
SD	Standard Deviation
SFAs	Saturated Fatty Acids
