# Supplementary material for: Validity of an AI-Assisted Dietary Recording Application for Family-Based Nutritional Management in Young Patients with Anorexia Nervosa
Source: Nutrients. 2026 Feb 23;18(4):708. doi: 10.3390/nu18040708 (PMC12942889; doi:10.3390/nu18040708)
Supplement: Supplementary file 1 [file nutrients-18-00708-s001.zip › Supplementary_File_Figure_S1.pdf]

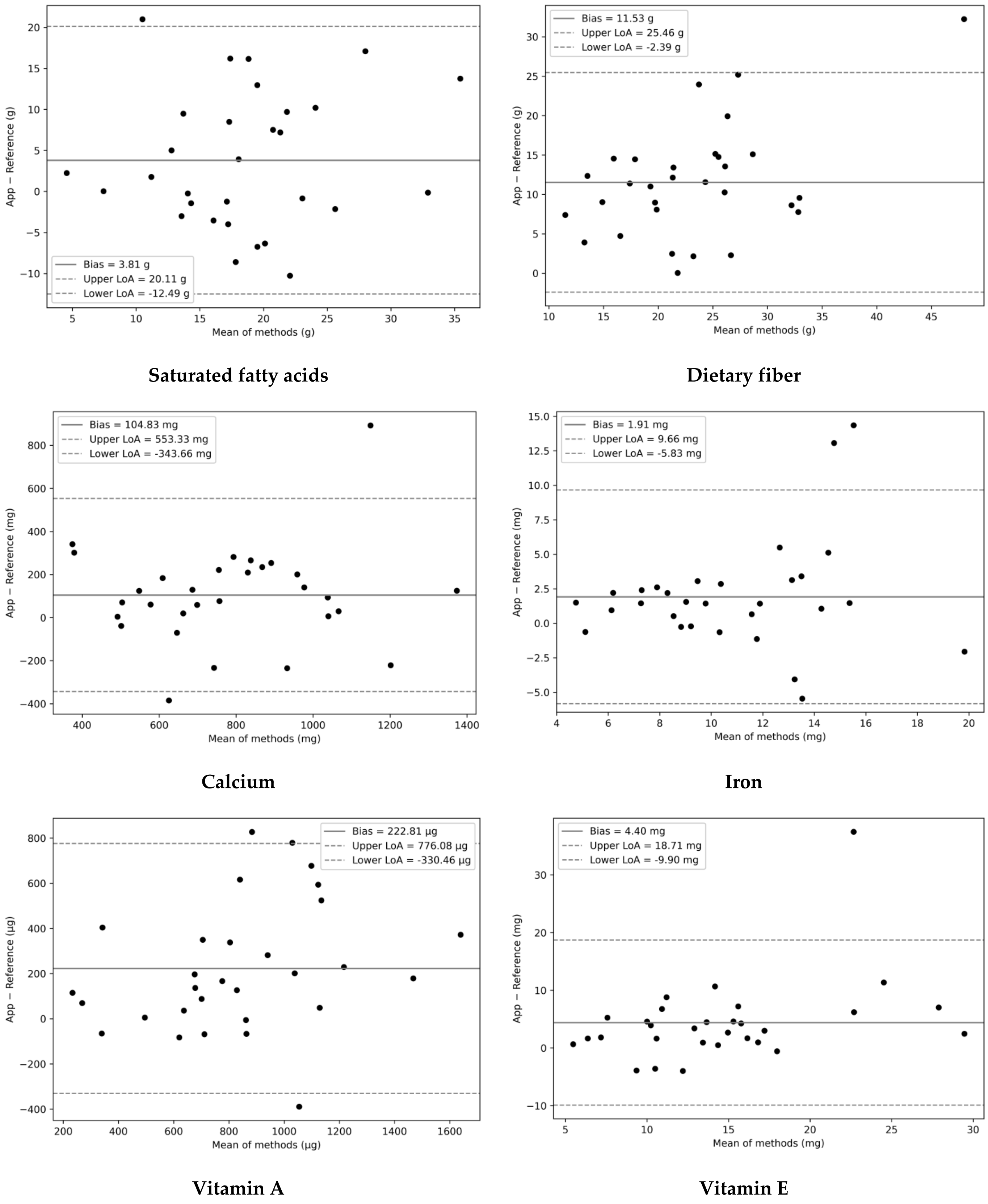

Figure S1. *Cont.*

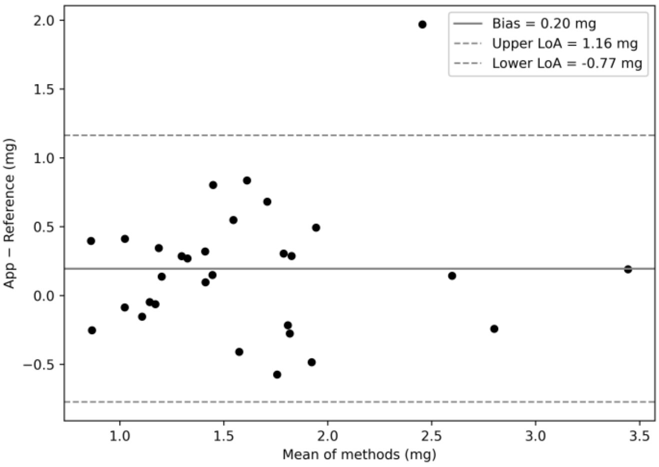

Vitamin B1

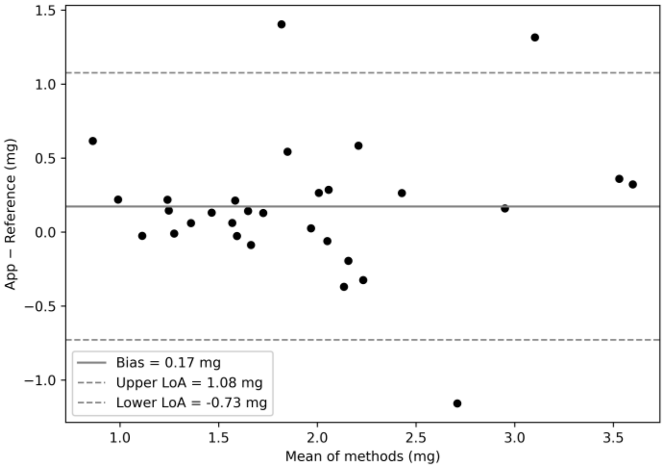

Vitamin B2

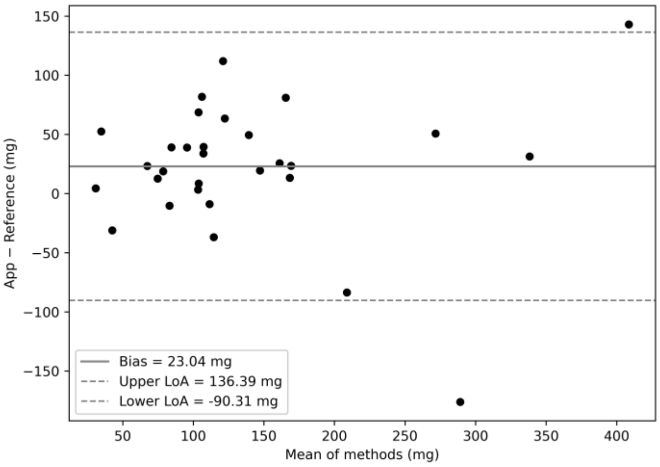

Vitamin C

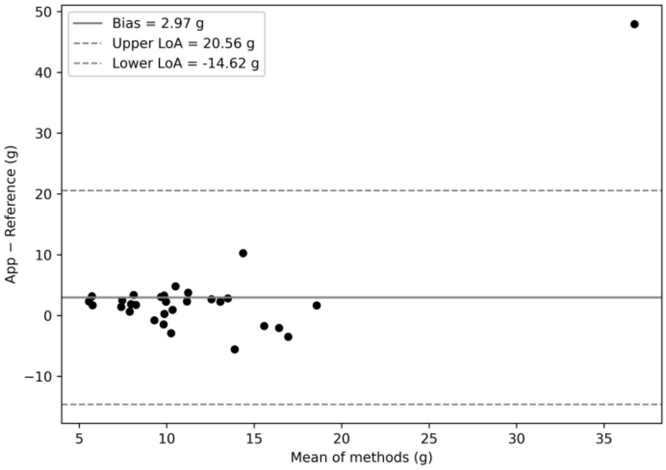

Sodium

Figure S1. Bland–Altman plots for nutrient intakes (saturated fatty acids, dietary fiber, calcium, iron, vitamins A, E, B1, B2, C, and sodium) comparing the app with the reference method (*n* = 30): The solid line indicates the bias, and the dashed lines represent the 95% limits of agreement (LoA).
