# Supplementary material for: Validity of an AI-Assisted Dietary Recording Application for Family-Based Nutritional Management in Young Patients with Anorexia Nervosa
Source: Nutrients. 2026 Feb 23;18(4):708. doi: 10.3390/nu18040708 (PMC12942889; doi:10.3390/nu18040708)
Supplement: Supplementary file 1 [file nutrients-18-00708-s001.zip › Supplementary_File_Figure_S2.pdf]

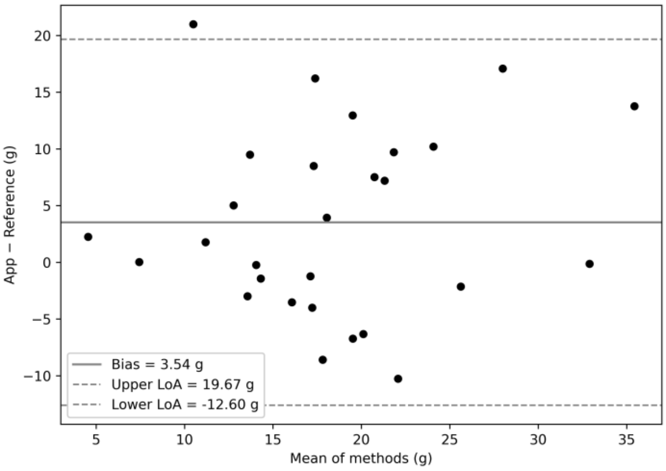

Saturated fatty acids

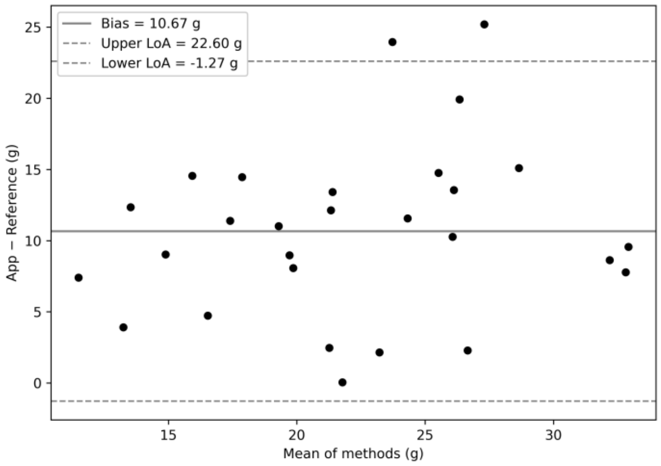

Dietary fiber

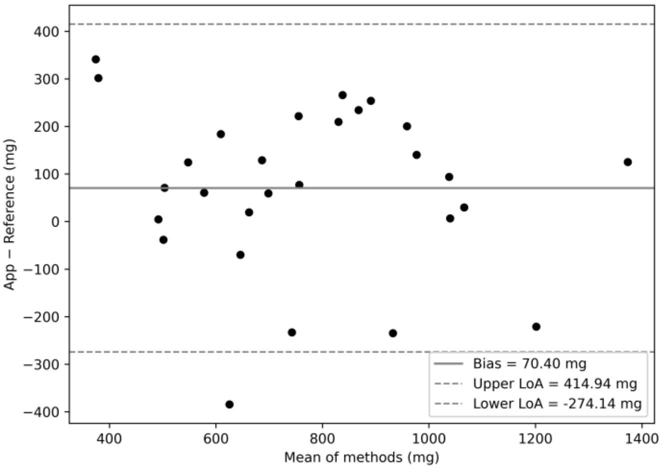

Calcium

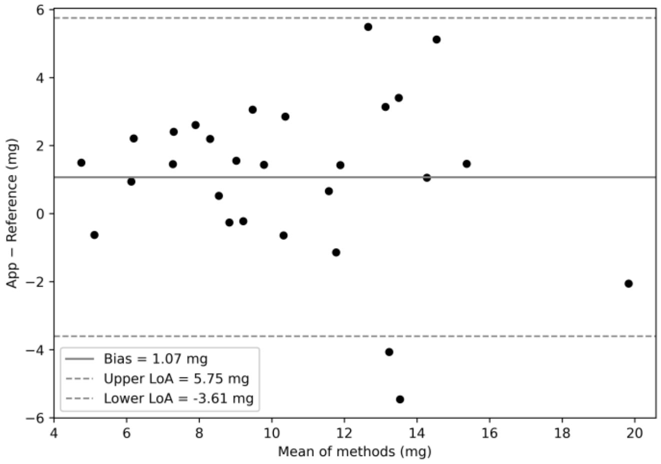

Iron

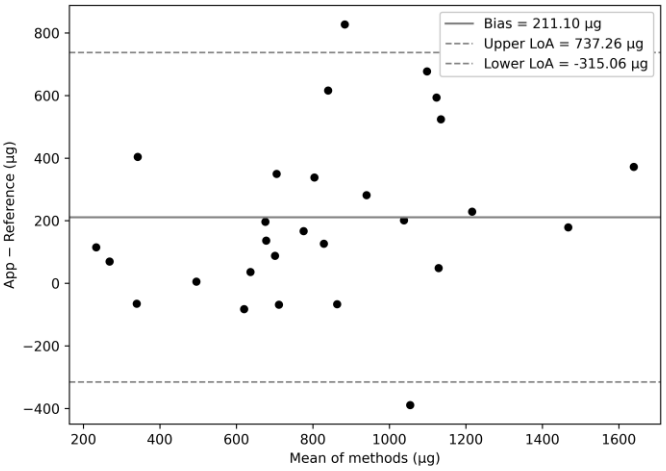

Vitamin A

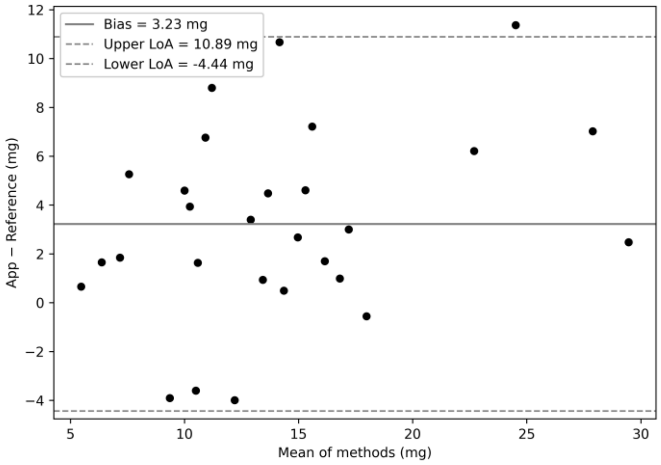

Vitamin E

Figure S2. *Cont.*

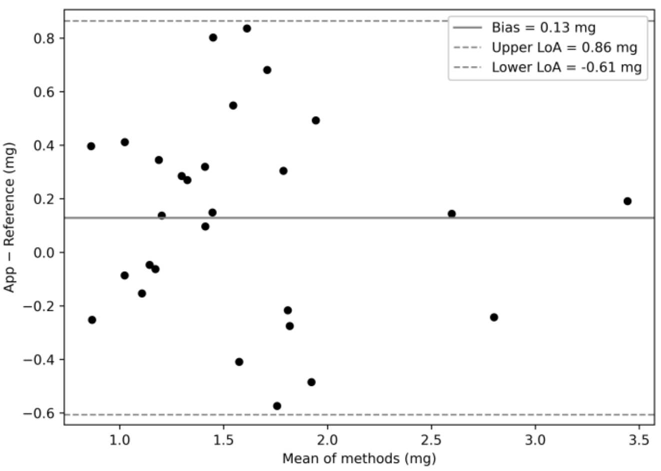

Vitamin B1

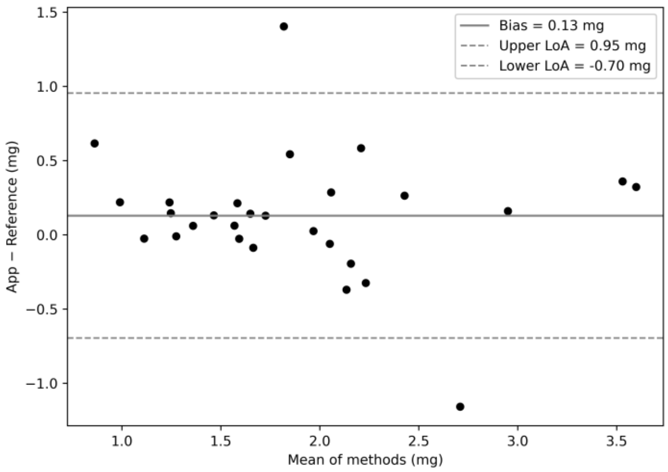

Vitamin B2

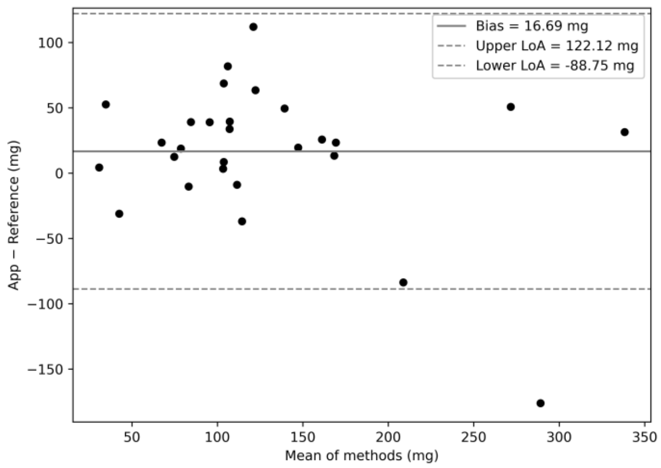

Vitamin C

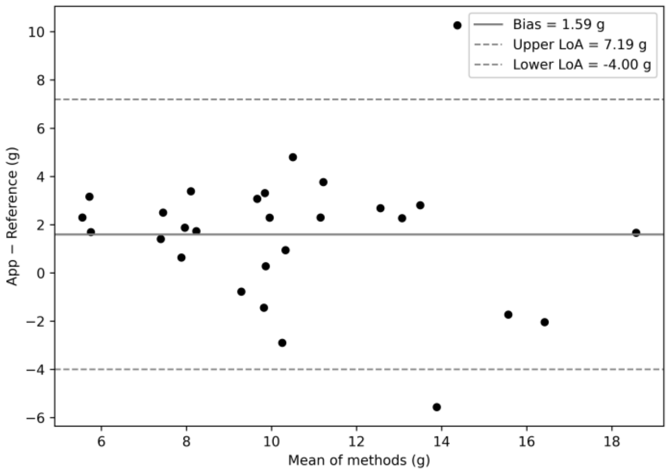

Sodium

Figure S2. Sensitivity analysis of Bland–Altman plots for nutrient intakes (saturated fatty acids, dietary fiber, calcium, iron, vitamins A, E, B1, B2, C, and sodium) comparing the app with the reference method (*n* = 28): The solid line indicates the bias, and the dashed lines represent the 95% limits of agreement (LoA).
